# Supplementary material for: Systemic GLP-1R agonist treatment reverses mouse glial and neurovascular cell transcriptomic aging signatures in a genome-wide manner
Source: Commun Biol. 2021 Jun 2;4:656. doi: 10.1038/s42003-021-02208-9 (PMC8172568; doi:10.1038/s42003-021-02208-9)
Supplement: Supplementary file 2 — Supplementary Information [file 42003_2021_2208_MOESM2_ESM.pdf]

Supplementary Information

Systemic GLP-1R agonist treatment reverses mouse glial and neurovascular cell transcriptomic aging signatures in a genome-wide manner

Li, Chen, Vong and Zhao *et al*.

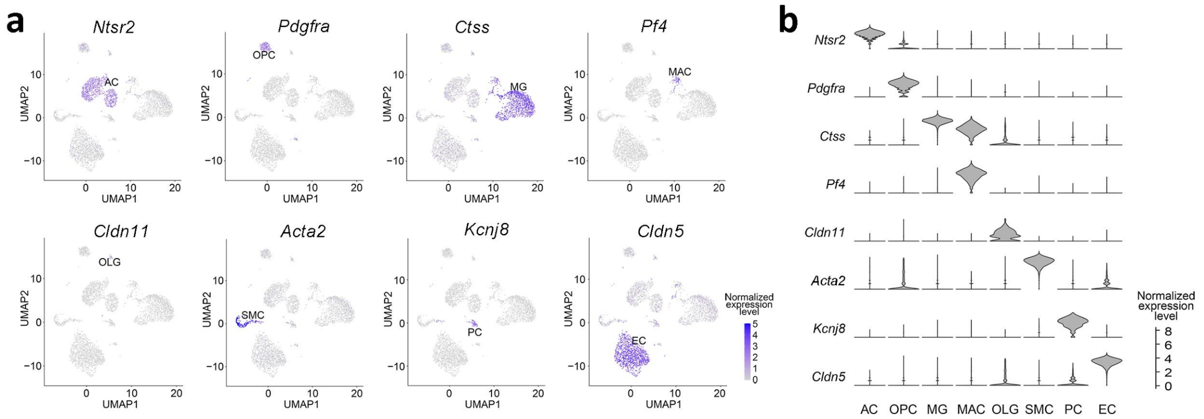

Supplementary Fig. 1. Primary cell type identification and their marker gene expression patterns. (a) UMAP visualization and **(b)** violin plots of cell type-specific marker gene expression patterns in the different cell type clusters. For clarity, 6000 cells were subsampled for visualization in each plot in **(a)**. Cell type abbreviations: same as in **Fig. 1**.

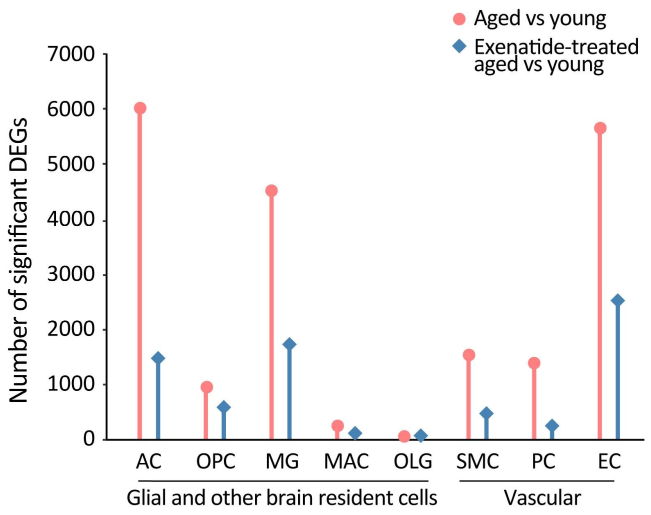

Supplementary Fig. 2. Number of significant differentially expressed genes (DEGs) for aged *vs* young adult mice, and exenatide-treated aged *vs* young adult mice comparisons in each cell type. Cell type abbreviations: same as in **Fig. 1**.

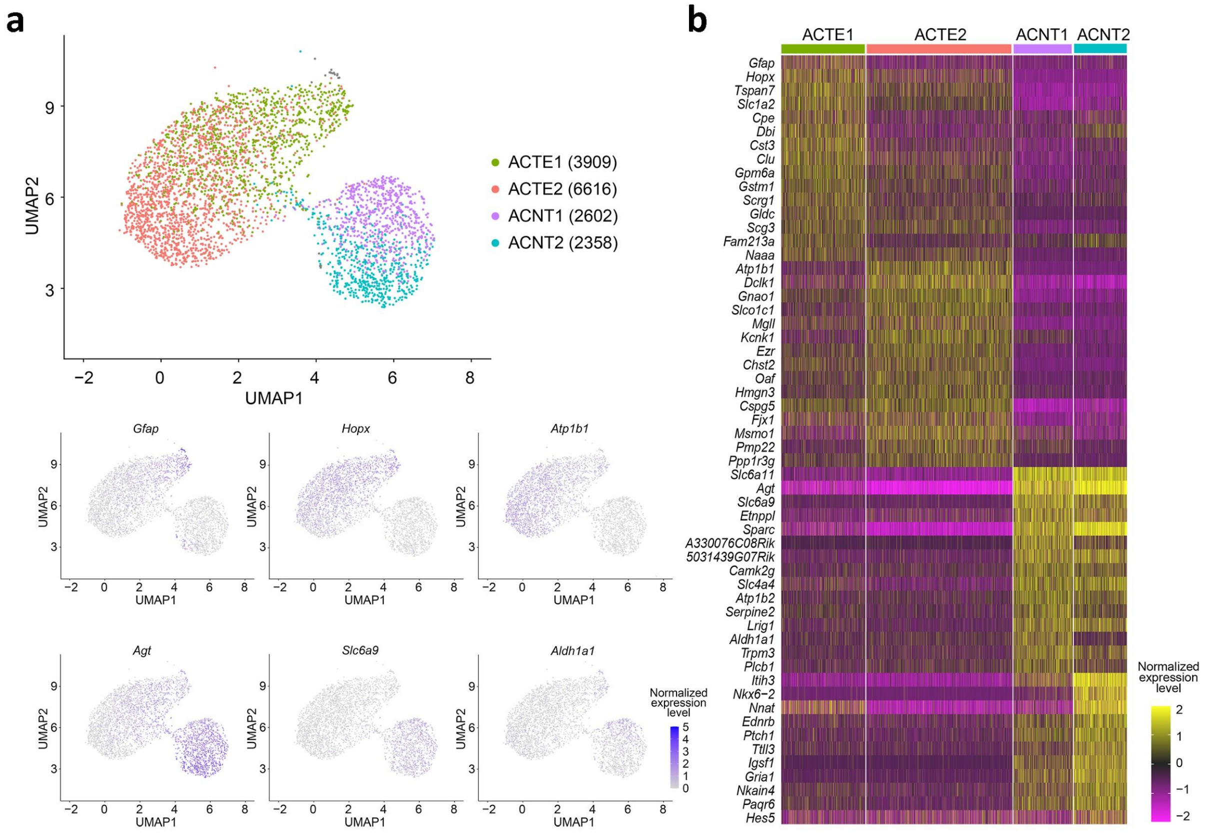

Supplementary Fig. 3. Identification of regional astrocyte (AC) subtypes. (a) UMAP visualization and **(b)** heatmap of marker gene expression patterns in the different regional AC subtype clusters, including telencephalic AC cluster 1 (ACTE1), telencephalic AC cluster 2 (ACTE2), non-telencephalic AC cluster 1 (ACNT1) and non-telencephalic AC cluster 2 (ACNT2). Numbers in brackets: cell numbers for the respective AC subtypes in the dataset. For clarity, 4000 cells were subsampled for visualization in each plot in **(a)**.

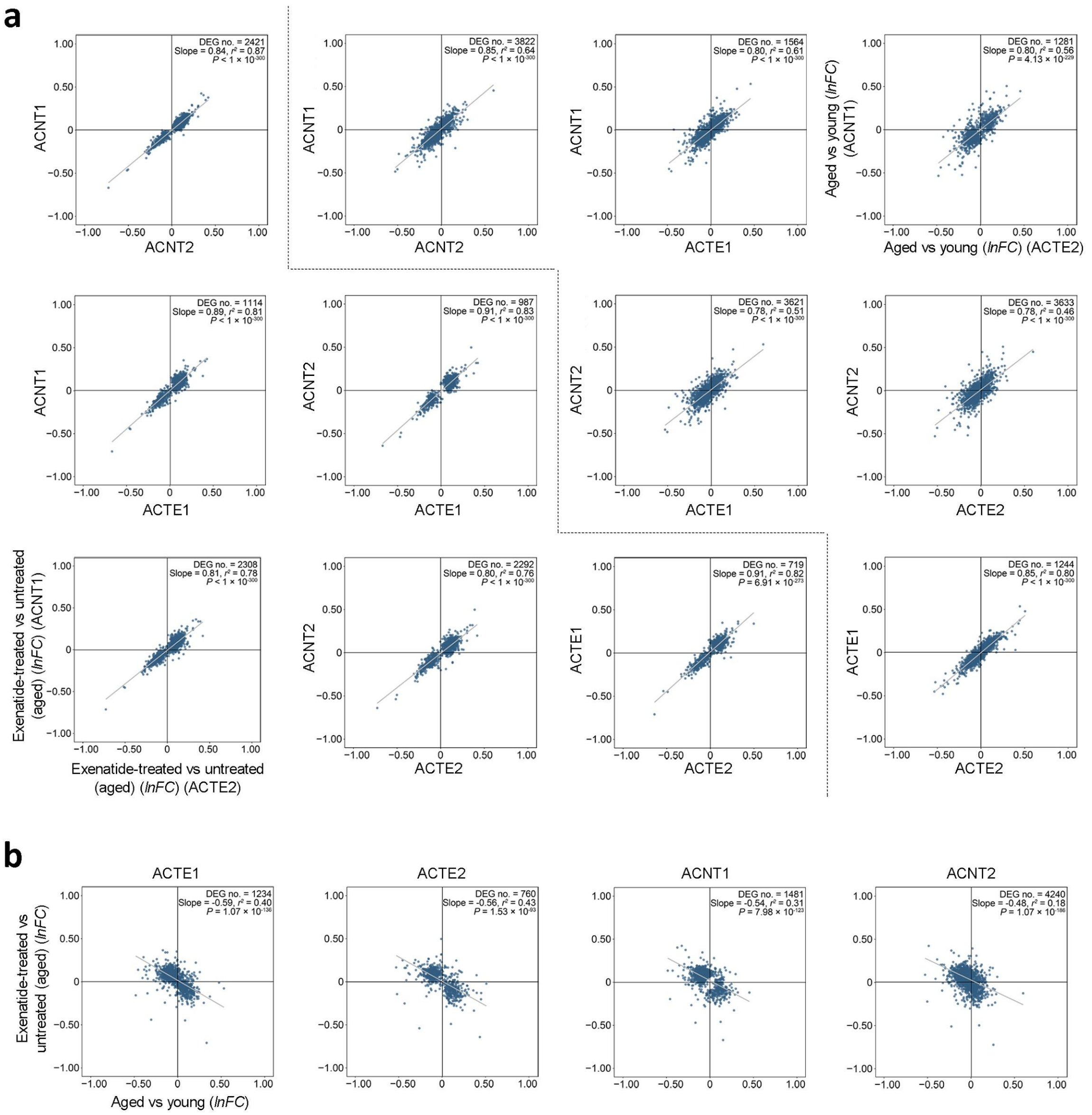

Supplementary Fig. 4. Consistency of differential expressions in the different regional astrocyte (AC) subtype clusters in aging and after GLP-1RA treatment. (a) Pairwise comparisons of age-related (above-diagonal plots in the array) and exenatide treatment-associated (below-diagonal plots in the array) differential expressions in the four AC subtypes (i.e., ACTE1, ACTE2, ACNT1 and ACNT2). **(b)** Expression changes in the regional AC subtypes in aging (x-axis) plotted against that after exenatide treatment (y-axis). For all plots in **(a)** and **(b)**: Each dot represents one differentially expressed gene (DEG). *lnFC*: natural log of fold change. Grey lines: lines of best fit by linear regression. Cell type / subtype abbreviations: same as in **Supplementary Fig. 3**.

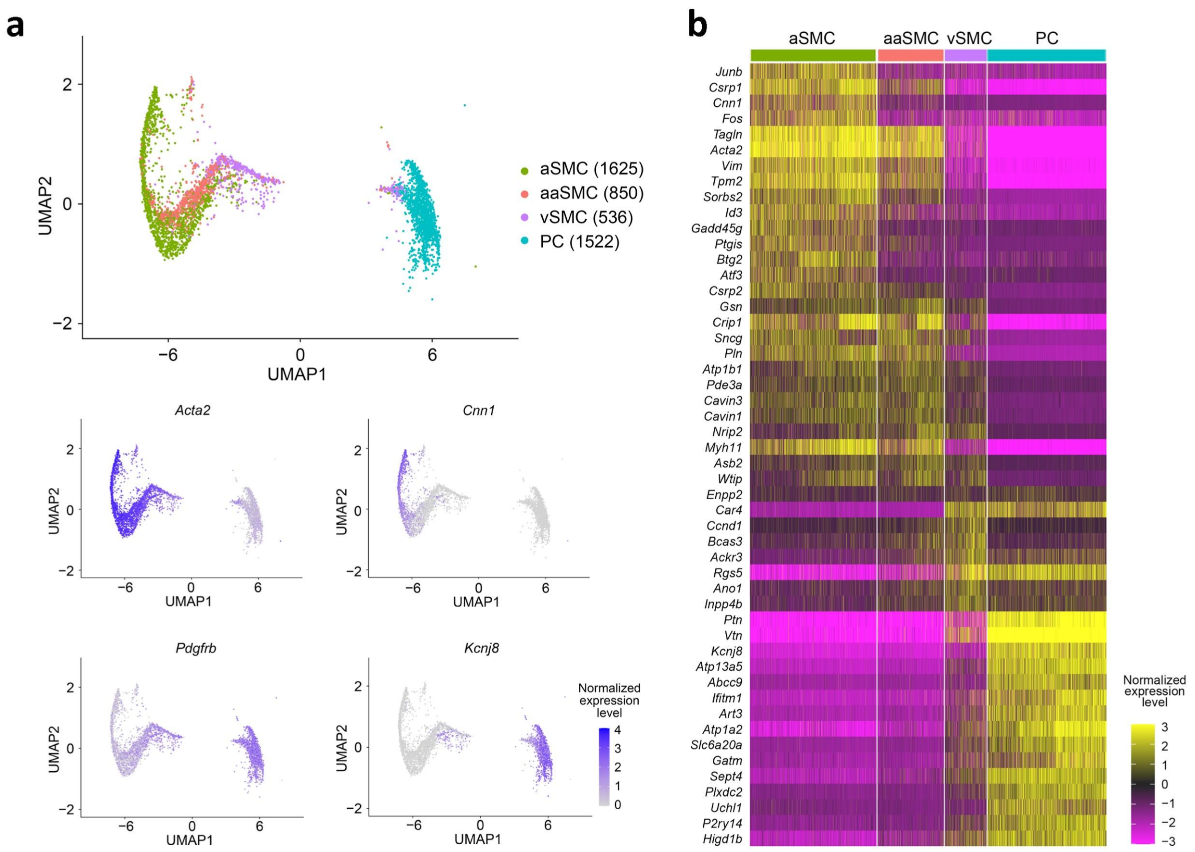

Supplementary Fig. 5. Identification of mural cell subtypes. (a) UMAP visualization and **(b)** heatmap of marker gene expression patterns in the different mural subtypes, including arterial smooth muscle cell (aSMC), arteriolar SMC (aaSMC), venous SMC (vSMC) and pericyte (PC). Numbers in brackets: cell numbers for the respective cell subtypes.

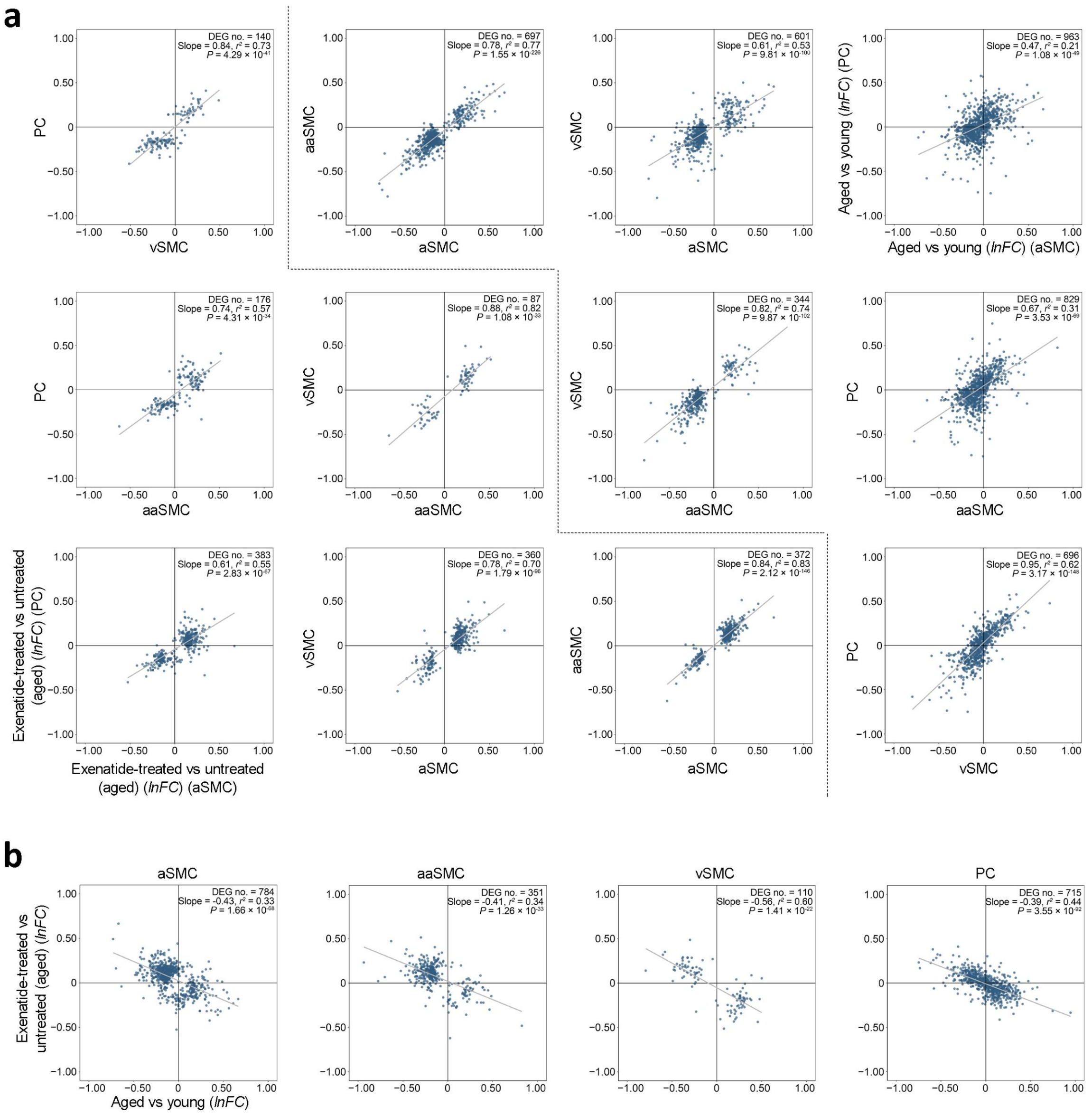

Supplementary Fig. 6. Consistency of differential expressions in the different mural cell subtypes in aging and after GLP-1RA treatment. (a) Pairwise comparisons of age-related (above-diagonal plots in the array) and exenatide treatment-associated (below-diagonal plots in the array) differential expressions in the four mural cell subtypes (aSMC, aaSMC, vSMC and PC). **(b)** Expression changes in the mural cell subtypes in aging (*x*-axis) plotted against that after exenatide treatment (*y*-axis). For all plots in **(a)** and **(b)**: Each dot represents one differentially expressed gene (DEG). *lnFC*: natural log of fold change. Grey lines: lines of best fit by linear regression. Cell type / subtype abbreviations: same as in **Supplementary Fig. 5**.

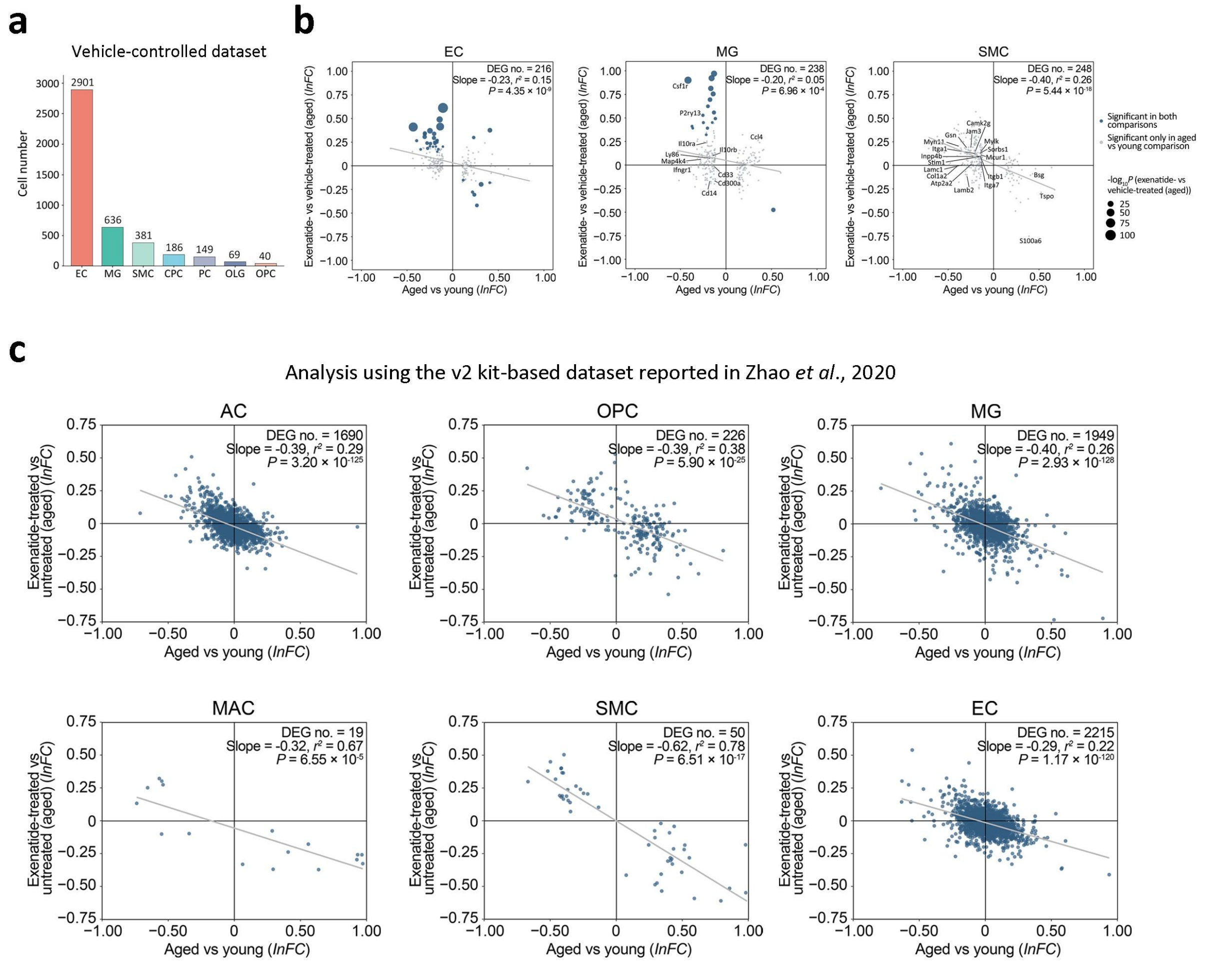

Supplementary Fig. 7. Further verification of main results on the genome-wide reversal of age-related transcriptomic changes by GLP-1RA treatment with two additional datasets. (a) Number of cells for each cell type in the vehicle-controlled dataset. **(b)** Age-related expression changes for the top reversed differentially expressed genes (DEGs) (*x*-axis, in the v3 kit-based dataset, see **Methods**) plotted against post-exenatide treatment expression changes (*y*-axis, in the vehicle-controlled dataset), for the cell types with top three cell numbers in the vehicle-controlled dataset (i.e., EC, MG and SMC). Each dot represents one DEG, with dot size scaling according to the negative logarithm of the *P*-values in the exenatide- *vs* vehicle-treatment comparison. *lnFC*: natural log of fold change. Grey lines: lines of best fit by linear regression. **(c)** Additional results of age-related transcriptomic changes reversed by exenatide treatment, based on analyzing a previously reported dataset (in Zhao *et al*., 2020, designated as v2 kit-based dataset as it was acquired using the 10X Genomics Chromium Single Cell 3' Reagent Kits v2). Identical analysis was performed as shown in **Fig. 1c** for the different cell types. Cell type abbreviations: same as in **Fig. 1**.
