# Supplementary material for: Systemic GLP-1R agonist treatment reverses mouse glial and neurovascular cell transcriptomic aging signatures in a genome-wide manner
Source: Commun Biol. 2021 Jun 2;4:656. doi: 10.1038/s42003-021-02208-9 (PMC8172568; doi:10.1038/s42003-021-02208-9)
Supplement: Supplementary file 3 — Description of Additional Supplementary Files [file 42003_2021_2208_MOESM3_ESM.pdf]

Description of Additional Supplementary Files

File name: Supplementary Data 1

Description: Source data underlying the graphs and charts presented in the main figures.
