# Supplementary material for: Systemic GLP-1R agonist treatment reverses mouse glial and neurovascular cell transcriptomic aging signatures in a genome-wide manner
Source: Commun Biol. 2021 Jun 2;4:656. doi: 10.1038/s42003-021-02208-9 (PMC8172568; doi:10.1038/s42003-021-02208-9)
Supplement: Supplementary file 5 — Reporting Summary [file 42003_2021_2208_MOESM5_ESM.pdf]

nature research

Corresponding author(s): Ho Ko

Last updated by author(s): Apr 28, 2021

Reporting Summary

Nature Research wishes to improve the reproducibility of the work that we publish. This form provides structure for consistency and transparency in reporting. For further information on Nature Research policies, see our Editorial Policies and the Editorial Policy Checklist.

Statistics

For all statistical analyses, confirm that the following items are present in the figure legend, table legend, main text, or Methods section.

n/a	Confirmed	
☐	☒	The exact sample size (*n*) for each experimental group/condition, given as a discrete number and unit of measurement
☐	☒	A statement on whether measurements were taken from distinct samples or whether the same sample was measured repeatedly
☐	☒	The statistical test(s) used AND whether they are one- or two-sided<br>*Only common tests should be described solely by name; describe more complex techniques in the Methods section.*
☐	☒	A description of all covariates tested
☐	☒	A description of any assumptions or corrections, such as tests of normality and adjustment for multiple comparisons
☐	☒	A full description of the statistical parameters including central tendency (e.g. means) or other basic estimates (e.g. regression coefficient) AND variation (e.g. standard deviation) or associated estimates of uncertainty (e.g. confidence intervals)
☐	☒	For null hypothesis testing, the test statistic (e.g. *F*, *t*, *r*) with confidence intervals, effect sizes, degrees of freedom and *P* value noted<br>*Give P values as exact values whenever suitable.*
☒	☐	For Bayesian analysis, information on the choice of priors and Markov chain Monte Carlo settings
☒	☐	For hierarchical and complex designs, identification of the appropriate level for tests and full reporting of outcomes
☐	☒	Estimates of effect sizes (e.g. Cohen's *d*, Pearson's *r*), indicating how they were calculated

Our web collection on statistics for biologists contains articles on many of the points above.

Software and code

Policy information about availability of computer code

Data collection	For mouse brain cell RNA sequencing, the raw fastq files were aligned by Cell Ranger (v3.0.0, 10X Genomics, US) and converted to count matrix for further processing.
Data analysis	The sequencing data processing were performed with the Seurat (v3.1.5) package and custom scripts in R (v3.6.1).<br>For differential expression analysis, the MAST (v1.8.2) package was used (which was already incorporated in the Seurat package).<br>Figures were generated by the Seurat package, the ggplot2 (3.3.1) package, and custom scripts in R (v3.6.1).).<br>Statistical tests and linear regression analysis were carried out in R (v3.6.1).

For manuscripts utilizing custom algorithms or software that are central to the research but not yet described in published literature, software must be made available to editors and reviewers. We strongly encourage code deposition in a community repository (e.g. GitHub). See the Nature Research guidelines for submitting code & software for further information.

Data

Policy information about availability of data

All manuscripts must include a data availability statement. This statement should provide the following information, where applicable:

- Accession codes, unique identifiers, or web links for publicly available datasets
- A list of figures that have associated raw data
- A description of any restrictions on data availability

The single-cell RNA sequencing data presented in the main figures has been deposited at the Broad Institute Single Cell Portal and are accessible at the following URL: [https://singlecell.broadinstitute.org/single_cell/study/SCP1182/glp1ra-brain-aging-reversal]. The dataset reported in our previous study (Zhao et al.) is accessible at the following URL: [https://singlecell.broadinstitute.org/single_cell/study/SCP829/aging-mouse-brain-kolab].

Field-specific reporting

Please select the one below that is the best fit for your research. If you are not sure, read the appropriate sections before making your selection.

☒ Life sciences ☐ Behavioural & social sciences ☐ Ecological, evolutionary & environmental sciences

For a reference copy of the document with all sections, see nature.com/documents/nr-reporting-summary-flat.pdf

Life sciences study design

All studies must disclose on these points even when the disclosure is negative.

Sample size	For the v3 kit-based dataset, we used 3 mice per group (young adult, aged and exenatide-treated aged groups) for the scRNA-seq experiments. For the vehicle-controlled dataset, we used 3 mice per group (vehicle- and exenatide-treated aged groups) for the scRNA-seq experiments. No prior sample size calculation was performed. Selection of sample sizes was based on past research experience judging potential biological effects relative to expected variability, typically requiring 3 animals per group for the type of experiments carried out.
Data exclusions	For the astrocyte single-cell transcriptomes, we focused on telencephalic and non-telencephalic astrocytes. We did not analyze a relatively small number of midbrain astrocytes (i.e. small sample size compared to telencephalic and non-telencephalic astrocytes, whereby midbrain astrocytes constituted <1% of the total number of astrocytes sampled).
Replication	The sequencing data presented came from batches of experiments with multiple animal subjects used for each group. All attempts to replicate the findings with independent batches of experiments were successful (see main text and Supplementary Fig. 7).
Randomization	Not required. This study involved comparison across age and treatment groups, and therefore samples were grouped by age and treatment as the primary distinguishing parameters.
Blinding	Not required. This study did not involve subjective measurements as all sequencing data were included with identical and strict criteria, and analyzed by identical software or programming pipelines.

Reporting for specific materials, systems and methods

We require information from authors about some types of materials, experimental systems and methods used in many studies. Here, indicate whether each material, system or method listed is relevant to your study. If you are not sure if a list item applies to your research, read the appropriate section before selecting a response.

Materials & experimental systems

n/a	Involved in the study
☒	☐ Antibodies
☒	☐ Eukaryotic cell lines
☒	☐ Palaeontology and archaeology
☐	☒ Animals and other organisms
☒	☐ Human research participants
☒	☐ Clinical data
☒	☐ Dual use research of concern

Methods

n/a	Involved in the study
☒	☐ ChIP-seq
☒	☐ Flow cytometry
☒	☐ MRI-based neuroimaging

Animals and other organisms

Policy information about studies involving animals; ARRIVE guidelines recommended for reporting animal research

Laboratory animals	Male C57BL/6J mice aged 2-3 months old and 18-20 months old, provided by the the Laboratory Animal Service Center of the Chinese University of Hong Kong and maintained at controlled temperature (22 – 23°C) with an alternating 12-hour light/dark cycle with free access to standard mouse diet and water. The ambient humidity was maintained at < 70 % relative humidity.
Wild animals	The study did not involve wild animals.
Field-collected samples	The study did not use samples collected from the field.
Ethics oversight	All experimental procedures were approved in advance by the Animal Research Ethical Committee of the Chinese University of Hong Kong and were carried out in accordance with the Guide for the Care and Use of Laboratory Animals.

Note that full information on the approval of the study protocol must also be provided in the manuscript.
